# Semiautomatic mapping of a national drug terminology to standardised OMOP drug concepts using publicly available supplementary information

**DOI:** 10.1186/s12874-025-02669-0

**Published:** 2025-09-26

**Authors:** Florian Katsch, Ágota Mészáros, Tibor Héja, Rada Hussein, Georg Duftschmid

**Affiliations:** 1 Institute of Medical Information Management, Center for Medical Data Science, Medical University of Vienna, Vienna, Austria; 2 Ludwig Boltzmann Institute for Digital Health and Prevention, Salzburg, Austria; 3 Institute for Clinical Data Management, Semmelweis University, Budapest, Hungary; 4 Faculty of Pharmacy, Center for Health Technology Assessment and Pharmacoeconomic Research, University of Pécs, Pécs, Hungary

**Keywords:** Drug terminology mapping, OMOP, CDM, RxNorm, Usagi, Health data standardisation, Secondary use of health data

## Abstract

**Background:**

Mapping national drug terminologies to internationally recognized standards is essential for harmonising health data across regions and supporting secondary data use. In Austria, the national drug terminology lacks fine-granular mappings to RxNorm and RxNorm Extension (RxN/E), limiting its integration into the Observational Medical Outcomes Partnership (OMOP) Common Data Model (CDM). This study aims to semiautomatically map Austria’s national drug terminology to RxN/E, to enable improved interoperability and data standardisation for secondary use.

**Methods:**

We implemented a semiautomated mapping approach using public supplementary data to bridge the gap between national drug concepts and RxN/E concepts. Probabilistic matching and hierarchical refinement techniques were applied to derive finer-grained and more meaningful mappings than previously available ingredient level mappings via the Anatomical Therapeutic Chemical (ATC) classification. We linked our mappings to other available European drug mappings for a validation of our results.

**Results:**

Our process successfully mapped 18,390 (95.42%) of Austria’s 19,273 drug concepts to RxN/E, surpassing previous mappings that focused solely on ingredient-level relationships. Specifically, we mapped 73.65% of the concepts to more specific RxN/E targets, such as branded drug boxes and quantified clinical drugs. We identified multiple vocabulary inconsistencies, including duplications and erroneous relationships within RxN/E, which were documented for improvement. The results are disseminated as Usagi-formatted CSV files and HL7 FHIR ConceptMaps to encourage transparency, ease of use, and community-driven refinement.

**Conclusions:**

The presented mapping approach highlights the feasibility and utility of leveraging publicly available supplementary data to create mappings between national drug terminology and RxN/E. Our method yields fine-grained mappings, enabling precise and comprehensive drug data integration for secondary use.

**Supplementary Information:**

The online version contains supplementary material available at 10.1186/s12874-025-02669-0.

## Background

In the current healthcare landscape, the secondary use of health data is seen as a cornerstone for driving advancements in research, clinical decision-making, policy making and citizen engagement [1, 2]. Leveraging existing health data for purposes beyond their initial collection not only maximises the value of resources invested in healthcare but also facilitates insights into population health trends, treatment effectiveness, and adverse event detection [3, 4]. Central to this endeavour is the seamless integration and interoperability of diverse healthcare data sources, including electronic health records (EHRs) and administrative claims data.

Within this landscape, the standardisation of drug-related terminology plays a pivotal role. Drug ontologies or vocabularies serve as structured frameworks for categorising and representing medication information, enabling effective communication and data exchange across healthcare systems. However, achieving interoperability among these terminologies presents a formidable challenge due to variations in naming conventions, granularity levels, used languages and national particularities [5–7].

The Observational Medical Outcomes Partnership (OMOP) has addressed this challenge by designating RxNorm, published by the United States National Library of Medicine (NLM), as standard vocabulary for coding drug-related concepts. However, because this is a US-centred vocabulary, an extension, the RxNorm Extension (RxE), was introduced by OMOP. RxE extends the RxNorm terminology with internationally used drug concepts and attributes [8]. Mapping a national drug terminology to RxNorm and OMOP’s RxNorm Extension terminology enables harmonisation of drug-related data independent of national terminologies, and thus supports analysis across disparate healthcare datasets.

Manual mapping between different terminologies can be an error-prone, laborious process and usually involves domain experts. As such, there is interest in leveraging (semi-) automatic methods to facilitate terminology mapping and integration [9–11]. Additionally, semantic web technologies have been used in more formal ontology-based integration approaches [12, 13].

To circumvent these mapping efforts, the Anatomical Therapeutic Chemical (ATC) classification system is commonly used to bridge the gap between source drug terminologies and RxNorm, primarily due to its widespread availability and adoption. The ATC system categorises drugs on the basis of their anatomical target, therapeutic intent, and chemical properties, providing a hierarchical classification of active drug ingredients. However, ATC classifies drugs relatively coarse-grained, on the basis of substances instead of actual marketed drug products. As a result, a single drug product may fall into multiple ATC categories, reflecting its diverse pharmacological characteristics and therapeutic applications. Despite its limitations in granularity, ATC serves as a valuable resource for drug mappings and is often used either exclusively or in combination with other mapping efforts in OMOP projects due to existing ATC to RxNorm mappings within OMOP vocabularies [14–22].

Based on OMOP’s international drug vocabulary implementation process, this work aims to map Austria’s drug terminology to RxNorm and the RxNorm Extension by exploiting publicly available supplementary data. The approach first maps ingredients and dose forms, and then includes drug strength information, brands and package sizes. By exploiting RxNorm’s hierarchical structure, we are able to derive finer-grained mappings in comparison to ingredient level mappings obtained from relationships via ATC codes alone. Although this work focuses on mapping Austria’s drug terminology, the proposed methodology is applicable to other source terminologies as well and might benefit the broader research community.

To emphasise, this paper pursues the following objectives:

- map the national drug terminology to RxNorm and RxNorm Extension in a semiautomated process
- utilise publicly available supplemental data
- derive as fine-grained concepts as possible within RxNorm and RxNorm Extension
- validate and disseminate mapping results as a starting point for future discussion and refinement

## Methods and materials

### Source terminology

Austria’s national health information exchange (HIE) infrastructure requires medication data to be coded according to the national drug register code system (ASP-Liste, German acronym for “list of human medicinal products”, abbreviated as ASP in this paper) publicly available from the national terminology server [23]. This code system includes all human medicinal products marketed within the country (both with national registration and EU-wide registration), and holds among others, information about the name, registration status, ingredients, strengths and ATC references for 19,273 drug concepts. Information within this terminology was aggregated from various information providers, e.g. Austrian Agency for Health and Food Safety. All contained drugs are identified via a nationally unique identifier, the PZN (German acronym for central drug number). Note that the Austrian PZN is not equivalent to the German PZN.

These source data are augmented by information on drug box sizes and optional volume information from the Austria-Codex KHIX2 [24], a database containing detailed information on drug products for human use published by the Austrian Pharmacists’ Publishing House (Österreichischer Apotheker-Verlag). It contains a superset of ASP concepts with additional information on e.g. drug-drug-interactions in a highly structured, regularly updated and licensed XML database to be integrated into e.g. hospital information systems. We accessed this database through a license granted to the Medical University of Vienna. This database, although licensed, was needed since drug box size and volume information is missing for some concepts in ASP.

### Supplementary data

Additionally, supplementary publicly available datasets are used in this work to aid the mapping process. All OMOP vocabularies are obtained through the Athena tool (athena.ohdsi.org), a searchable database operated by OHDSI. Table 1 lists relevant the data sources used, a description of each and information on how these data sources were accessed.

**Table 1:** Relevant supplementary data sources for this project. See additional file 1 for a detailed description of the attributes we used from those data sources

Name	Content	Notes	Availability
OMOP CTD	Concepts from the Comparative Toxicogenomics Database (CTD) incorporated into OMOP’s vocabularies. Concepts use the CAS numbers (Chemical Abstract Service Registry Number) as code	Used to assign CAS numbers via OMOP’s “non standard to standard map” relationship to RxNorm and RxNorm Extension concepts of the ingredient concept class.	public - access via Athena [25]
OMOP ATC	Concepts from the World Health Organizations (WHO) ATC drug classification system. These concepts are augmented by OMOP with a variety of relationships to RxNorm and RxNorm Extension.	The “ATC - RxNorm pr lat” relationship is used to assign ATC codes to RxNorm and RxNorm Extension concepts of concept class ingredient and precise ingredient.	public - access via Athena [26]
Karapetian et al. terminology alignment of RxNorm and EDQM (EDQM-RX)	[27] contains a mapping from RxNorm Dose Forms to EDQM Dose Forms used for drugs described by the European Medicines Agency (EMA)	Used to assign RxN/E dose forms to EU drugs.	public - access via supplementary data in [27]
EMA - Product information URLs for member states (EMA-PI)	URLs to product information sheets for drug products recognized by EMA	Product information sheets are available as PDFs; EU drug registration numbers and EDQM dose forms are extracted from them. In combination with information from EDQM-RX, EU drugs can be assigned to RxN/E dose forms.	public - access via EMA website [28]
EMA - Substances Management Services (SMS)	Substance names in various European languages	Synonyms and translations of substance names share a common identifier; a preferred name in English language is designated. Used to derive synonyms and translations for ingredients.	public - access via EMA’s SPOR website [29]
Raw data of substance names (BEZVO)	Substance names required to be used in regulatory affairs in Germany	Contains, among other information, substance names in German language and assigned CAS registry numbers. Mainly used to translate German ingredients and to gather synonyms.	public - access via German Federal Institute for Drugs and Medical Devices [30]
Hungarian medicine identification number (TTT) to RxN/E mapping	Mapping of Hungarian medicines to RxNorm and RxNorm Extension, mapped by hand in 2021	Part of these Hungarian medicines have an EU drug registration number, through which they can be linked to Austrian PZN number. This relationship was used to validate our mapping results and was not used in the mapping process itself	private dataset, accessed from two of the authors (TH, AM)

### Target terminologies

**Fig. 1:**
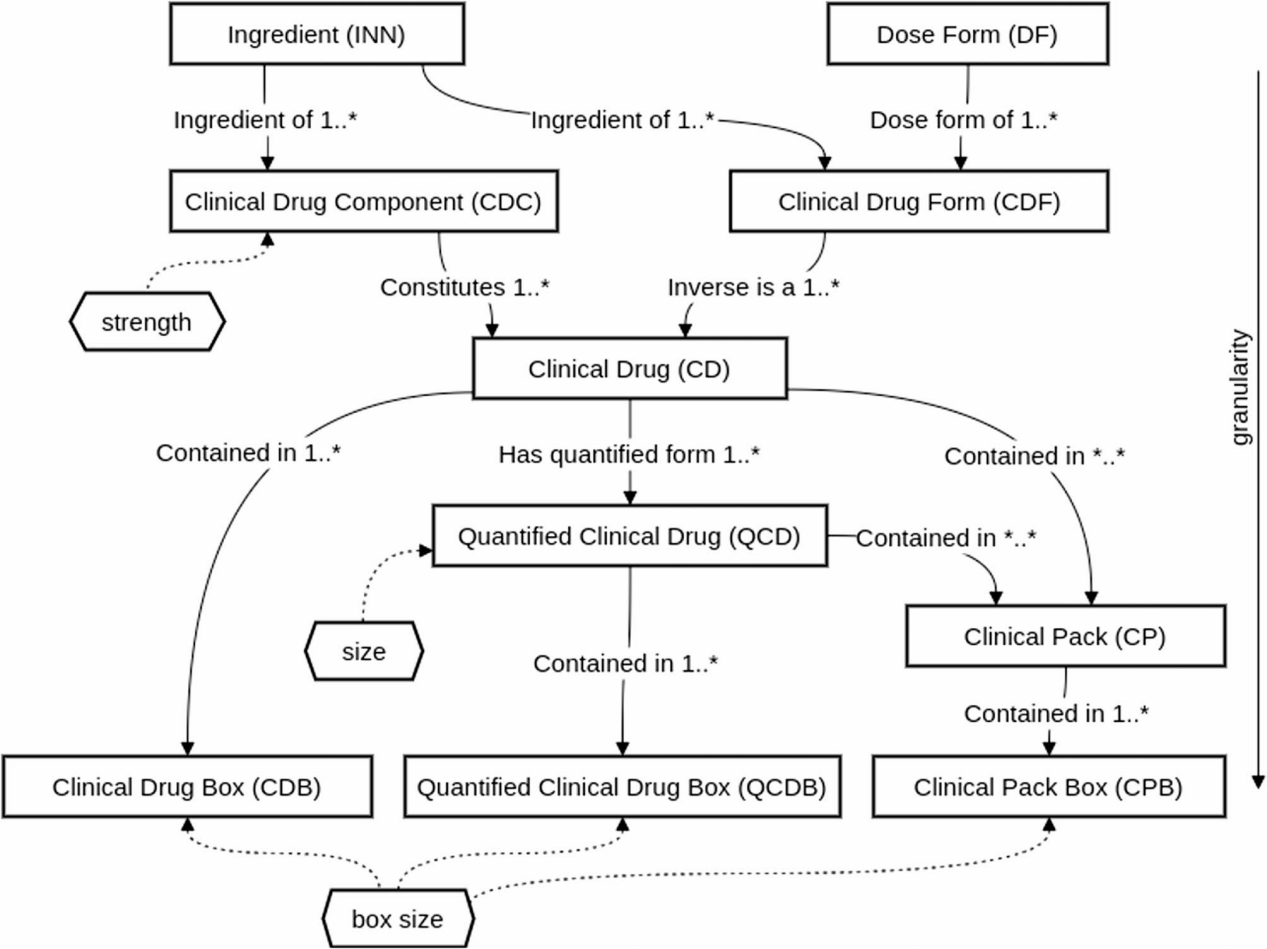
A selection of RxN/E concept classes, additional information (e.g. box size) and relationships between these classes as used within OMOP’s drug vocabulary

OMOPs standard vocabularies for drug related concepts are RxNorm and RxNorm Extension [8]. These vocabularies serve as the mapping targets for all other medication-related vocabularies within OMOP. The alignment and linking of different medication vocabularies to RxNorm and RxNorm Extension ensures consistent and interoperable representation of medication-related information. We consider the two vocabularies jointly in this work and therefore refer to them as RxN/E. Concepts within these vocabularies are hierarchically structured by linking concepts of certain concept classes (‘term types’ in NLM’s RxNorm) via concept relationships (‘relationships’ in NLM’s RxNorm). This hierarchical structure is a modified version of NLM’s RxNorm structure [8]. Figure 1 shows a selection of concept classes and their relationships relevant to this project. Refer to Table 2 for a textual description of those concept classes and examples.

OMOP’s RxNorm contains normalised names for drugs used in the US with links to other drug vocabularies. Originally published by the NLM, it includes over 200,000 hierarchically organised concepts e.g. for ingredients, drug forms and drug products used in clinical practice [31]. Since it is used as standard vocabulary for OMOP’s drug domain, a rich set of relationships to other vocabularies within OMOP exist. Some of the most prominent and often used relationships include the mapping of ATC codes to RxNorm’s ingredient concepts. OMOP’s RxNorm Extension augments and is integrated into the RxNorm vocabulary to incorporate internationally used drugs. It contains about 1.9 million concepts and notably also introduces additional concept classes for e.g. liquid formulations and drugs packaged in boxes. Both terminologies are publicly available and were accessed via OMOP’s Athena service [8, 32].

**Table 2:** A selection of rxn/e concept classes used in this work, ordered by increasing amount of information (granularity) they convey on drug products. Classes marked with * are considered as mapping targets, classes marked with ^B^ are also available as a version with additional information on the brand name

Concept Class	Coveys information on	Example
Ingredient*	Ingredient	penicillin G
Dose Form	Dose Form	Injectable Solution
Brand Name	Brand Name	Retacillin
Clinical Drug Component^B^	Ingredient, Strength	Penicillin G 59.4 MG/ML
Clinical Drug Form^B^	Ingredient, Dose Form	Penicillin G Injectable Solution
Clinical Drug*^B^	Ingredient, Strength, Dose Form	Penicillin G 59.4 MG/ML Injectable Solution
Clinical Drug Box*^B^	Ingredients, Strength, Dose Form, Box Size	Penicillin G 59.4 MG/ML Injectable Solution Box of 6
Quantified Clinical Drug*^B^	Ingredients, Strength, Dose Form, Size	2 ML Penicillin G 59.4 MG/ML Injectable Solution
Quantified Clinical Drug Box*^B^	Ingredients, Strength, Dose Form, Size, Box Size	2 ML Penicillin G 59.4 MG/ML Injectable Solution Box of 3
Clinical Pack*^B^	Clinical Drugs, their number	{(2 ML Penicillin G 15 MG/ML Injectable Solution)/(2 ML Penicillin G 59.4 MG/ML Injectable Solution) } Pack
Clinical Pack Box*^B^	Clinical Drugs, their number, Box Size	{(2 ML Penicillin G 15 MG/ML Injectable Solution)/(2 ML Penicillin G 59.4 MG/ML Injectable Solution) } Pack box of 3
Marketed Product	Ingredients, Strength, Dose Form, Supplier, and optionally information on Brand name, Size, Box Size, Pack content (Clinical Drug and their number)	{(2 ML Fibrinogen 70 MG/ML Injectable Solution)/(2 ML Thrombin 1000 UNT/ML Injectable Solution) } Pack [Evicel] by Johnson & Johnson

### Mapping approach

**Fig. 2:**
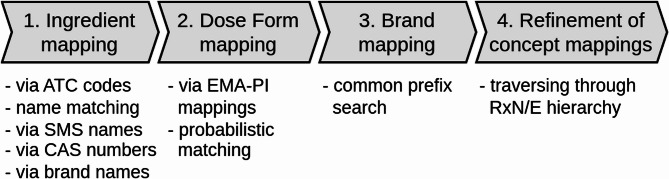
Overview of the proposed mapping methodology. Steps 1-3 seek to map ingredients, dose forms and brands, which are further refined in step 4

In this section, we outline our methodology used for mapping drug concepts from ASP to RxN/E. The approach is oriented on the hierarchical concept structure of RxN/E, starting from the most basic elements, such as ingredients, dose forms and brands, and progressively refining our mappings using, e.g., drug strength information (see Fig. 2). In contrast to OMOP’s “international drug vocabulary implementation process” [33], we do not aim at introducing new RxNorm Extension concepts but rather attempt to map to existing concepts within RxN/E.

*Mapping ingredients* forms the foundational step in our approach. This process starts by extracting ingredients from the source data as they are distributed across several fields. Ingredients are generally present in German language in the source vocabulary, although often those names are derived from Latin substance names or brands. RxN/E ingredients are generally present in English language. Since the distinction between active ingredients and excipients can be disputable, we only considered active ingredients, as designated by the source terminology. However, ASP itself provides valuable synonyms for ingredient names that can be used in the following steps. The sets of synonyms are further extended by generating trivial text variations, e.g., altering non-alphanumeric characters, for instance by replacing spaces with hyphens. The following mapping approaches are subsequently performed:

- ATC codes can be used to match ingredients in some instances, namely, where the source drug concept only has exactly one active ingredient and the corresponding ATC code itself only links to one RxN/E ingredient. For this purpose, we exploit OMOP’s “ATC - RxNorm primary lateral” relationships that link monocomponent ATC concepts with their semantically equivalent RxN/E ingredient concept. As an example, the ASP ingredient “Benzocain” is mapped to RxNorm’s “benzocaine” concept via the ATC code D04AB04 “benzocaine; topical”.
- Exact ingredient name matching involves comparing ingredient names and their synonyms to RxN/E ingredients to identify direct correspondences. E.g. the ASP ingredient “Olipudase Alfa” has a direct string match with the RxNorm ingredient “olipudase alfa”.
- Since source ingredient names are represented mostly in German language, translations to English are performed via EMA’s list of ingredients from the Substances Management Services (SMS). Translations are treated as synonyms and matched against RxN/E ingredients. E.g. the ASP ingredient “Salicylsäure” can be translated to “salicylic acid” which is available in RxNorm.
- Semantically equivalent chemical compounds are matched by comparing ingredient names and their synonyms against the BEZVO dataset, which contains substance names in German language and their corresponding CAS registry numbers. With this identifier a relationship to RxN/E ingredients can be obtained via the CTD dataset. OMOP’s CTD vocabulary links CTD concepts via the “maps to” relationship to RxN/E ingredients. E.g. the ASP ingredient “Zinksulfat-Heptahydrat” was translated via the CAS number 7446-20-0 to the RxNorm ingredient “zinc sulfate”.
- RxN/E’s brand names are used to derive ingredient mappings in cases where all source drug concepts are assigned to a brand that has only one associated active ingredient. E.g. the brand name “Digimerck” is directly related to only one RxNorm ingredient “digitoxin”, thus we assign ASP ingredients with matching brand name to this ingredient concept.

Utilising semantic correspondences across terminologies (e.g. BEZVO via CAS via CTD) enables the identification of candidate matches even when there are slight differences in naming conventions and languages between the two vocabularies. In some cases, the ingredient mapping step produces a set of candidate target concepts, which are further refined in subsequent steps. If an ingredient mapping is already given by the ATC relation, this existing mapping is in any case respected and refined by this method where applicable.

*Dose Form mapping*, is not straightforward, since dose forms are represented highly dissimilar across drug vocabularies [33]. suggested mapping dose forms of source drug vocabularies to multiple RxN/E dose forms, to account for differences in concept granularity. Furthermore, dose forms are named in German language within the source vocabulary. Our method uses the following two approaches to derive a dose form mapping:

- EU drug registration numbers are extracted from the source vocabulary, and their product information sheet is retrieved via the EMA-PI dataset. Since these product information sheets follow a common structure, the dose from information can easily be extracted and used to find exact matches in RxN/E’s dose form concepts.
- Probabilistic matching involves collecting hints for dose form mappings from all source drug concepts, with the intention of later predicting the most likely dose form mapping. For a given drug, we use its already mapped ingredients, ATC codes, and brand name to retrieve all the dose forms of drug concepts within RxN/E that match these concepts. Every source drug concept contributes to a drug form mapping by either “voting” for a single dose form mapping or dividing its vote among a set of dose form mappings if multiple were retrieved. Once mappings to (branded) drug components are established (see below), dose forms of all matching drug components are also gathered as votes. After all possible votes are collected, the target dose forms are determined by evaluating their accumulated weighted votes. As a consequence, one source dose form can be assigned to multiple RxN/E dose forms, with the potential for further refinement in subsequent steps.

*Brand mapping* is done by performing a longest common prefix search between the source drug name and RxN/E brand names. The input strings are tokenized using all non-alphanumeric characters as boundaries to enhance performance and robustness. This approach is used since no dedicated attribute for brand name is available within ASP and therefore brand name information must be extracted from the concept name.

*Quantity information* is available from the source vocabulary as a numerical value together with a unit descriptor at the ingredient level (strength). For some source drug concepts, the number of e.g. oral tablets sold as a packaged product is available (box size). However, this information alone is not sufficient to derive the quantity of a drug product with a liquid formulation or content expressed as the number of actuations for dosed inhalers. Therefore, additional size and box content information is extracted from the licensed KHIX2 vocabulary. All quantities are converted to RxN/E normalised quantities (e.g. RxN/E uses milligrams [mg] as default unit for weights).

*Refinement of concept mappings:* By traversing through the RxN/E hierarchy, we continuously refine our mappings on the basis of the available data and the hierarchical relationships within RxN/E. This iterative refinement is oriented along RxN/E’s structure and principle relationships depicted in Fig. 1:

- Clinical Drug Component (CDC) mappings are derived from already mapped ingredients by integrating their strength information. Due to ambiguous strength information or ingredient mappings to multiple RxN/E concepts, multiple possible CDC mappings might be derived in this step. Optionally, brand mapping is used to assign a Branded Drug Component (BDC) concept. In turn, this BDC mapping can be used to narrow down CDC mappings from the previous step, thus further refining the mapping. This strategy of gathering all plausible mappings to refine them in subsequent steps is applied likewise in the following steps.
- Clinical Drug Forms (CDF) are used implicitly to generate mappings to Clinical Drugs (CD) by combining all already mapped CDCs with the mapped dose form. A CD is constituted by a number of CDCs and CDFs corresponding to the number of ingredients a drug product has. Brand information is again used to refine the CD mapping and derive a Branded Drug (BD) mapping
- Drugs in liquid form or gases are represented within RxN/E as Quantified Clinical Drugs (QCD) or Quantified Branded Drugs (QBD), and a mapping can thus be derived by incorporating the size information
- The RxNorm Extension, in contrast to RxNorm, contains concept classes to capture the box size of a marketed drug product. Most drug products outside the US are sold as prepackaged boxes [32]. The incorporation of box sizes allows the retrieval of Clinical Drug Box (CDB), Quantified Clinical Drug Box (QCDB) and Clinical Pack Box (CPB) mappings along with their branded counterparts

This mapping methodology ensures that ingredient level mappings via ATC codes are used as a baseline and, at the very same time, realises the potential for refinements to eventually derive more detailed and thus valuable drug mappings.

### Implementation

The implementation follows the pipe and filter architecture by incrementally altering the input concepts to derive RxN/E mappings. Implemented as an open source Python project, it relies on a local SQLite OMOP database populated with the vocabularies mentioned in Table 1. The various supplementary data sources are gathered and cached upon first execution. The implementation outputs a Usagi-styled CSV file for seamless transition to OMOP’s vocabulary mapping tool Usagi. Optionally, a Usagi-styled CSV file can also be used as input, to preserve already approved mappings on repeated executions. Furthermore, we provide a Python tool to convert Usagi-styled CSV files to HL7 FHIR ConceptMap resources (versions R4B and R5). Implementations are publicly available at GitLab under the MIT licence [34, 35].

### Validation

To our knowledge, no previous drug mapping efforts have been made to link PZNs to RxN/E, apart from the aforementioned mapping via ATC codes. Due to these missing ground truth data and in an attempt to assess the accuracy and semantic consistency of the generated mappings, we rely on the EU drug registration numbers from the source vocabulary. They are used to link PZNs to Hungarian medicine identification number (TTT) concepts for which a mapping to RxN/E is available. We then compare the target RxN/E concepts for both mappings per EU drug registration number. The comparison is based on the notion of a common concept ancestor between two RxN/E concepts. For example, the two RxN/E clinical drug concepts 36888637 “Penicillin G 59.4 MG/ML Injectable Solution” and 44123049 “Penicillin G 1000000 UNT Injectable Solution” share the same clinical drug form concept 40072606 “penicillin G Injectable Solution” as a common ancestor. This comparison assesses the alignment of those two mappings. We introduce this common ancestor comparison since TTT was mapped primarily to concept of class “clinical drug box” and our approach tries to identify finer grained concepts and additionally includes brand information. Thus, a direct comparison based on target concept IDs is not feasible.

## Results

### Mapping results

In the course of our automatic mapping process we derived mappings for 18,390 (95.42%) of all 19,273 (100%) concepts contained in the source terminology. Further, 3,053 of the total 3,693 (82.66%) ingredient concepts found in the source vocabulary were mapped to RxN/E ingredients. A total of 5,079 (26.35%) source drug concepts could only be mapped to RxN/E ingredients, the baseline mapping via ATC references. The remaining 14,194 (73.65%) concepts could be mapped to a more specific RxN/E concept (see Table 3). Additionally, the process assigned RxN/E brand concepts to 11,917 (61.83%) source drug concepts. 92.89% of mappings have a single RxN/E concept as the target, 5.95% between two and five, and 1.16% more than five. We added manual mappings for 43 source concepts to RxN/E clinical packs, clinical pack boxes and their respective branded counterparts since the mapping of packs is not covered by our implementation (see discussion). Analysis of unmapped concepts revealed a disproportionate amount of concepts linked to the Level 2 ATC code B05, indicating that blood substitutes and perfusion solutions are particularly challenging for our mapping approach.

**Table 3:** Concept classes of mapping targets. Note that drug concepts mapped to multiple target concepts are included in these numbers

	Vocabulary		
Concept Class	RxNorm	RxNorm Extension	Total
*Unmapped*	-	-	883 (4.58%)
Ingredient	6579	1077	7656 (39.72%)
Clinical Drug	617	974	1591 (8.26%)
Branded Drug	170	653	823 (4.27%)
Clinical Drug Box	0	6240	6240 (32.38%)
Branded Drug Box	0	3936	3936 (20.42%)
Quantified Clinical Drug	22	150	172 (0.89%)
Quantified Branded Drug	50	105	155 (0.80%)
Quantified Clinical Drug Box	0	498	498 (2.58%)
Quantified Branded Drug Box	0	678	678 (3.52%)

### Validation

**Fig. 3:**
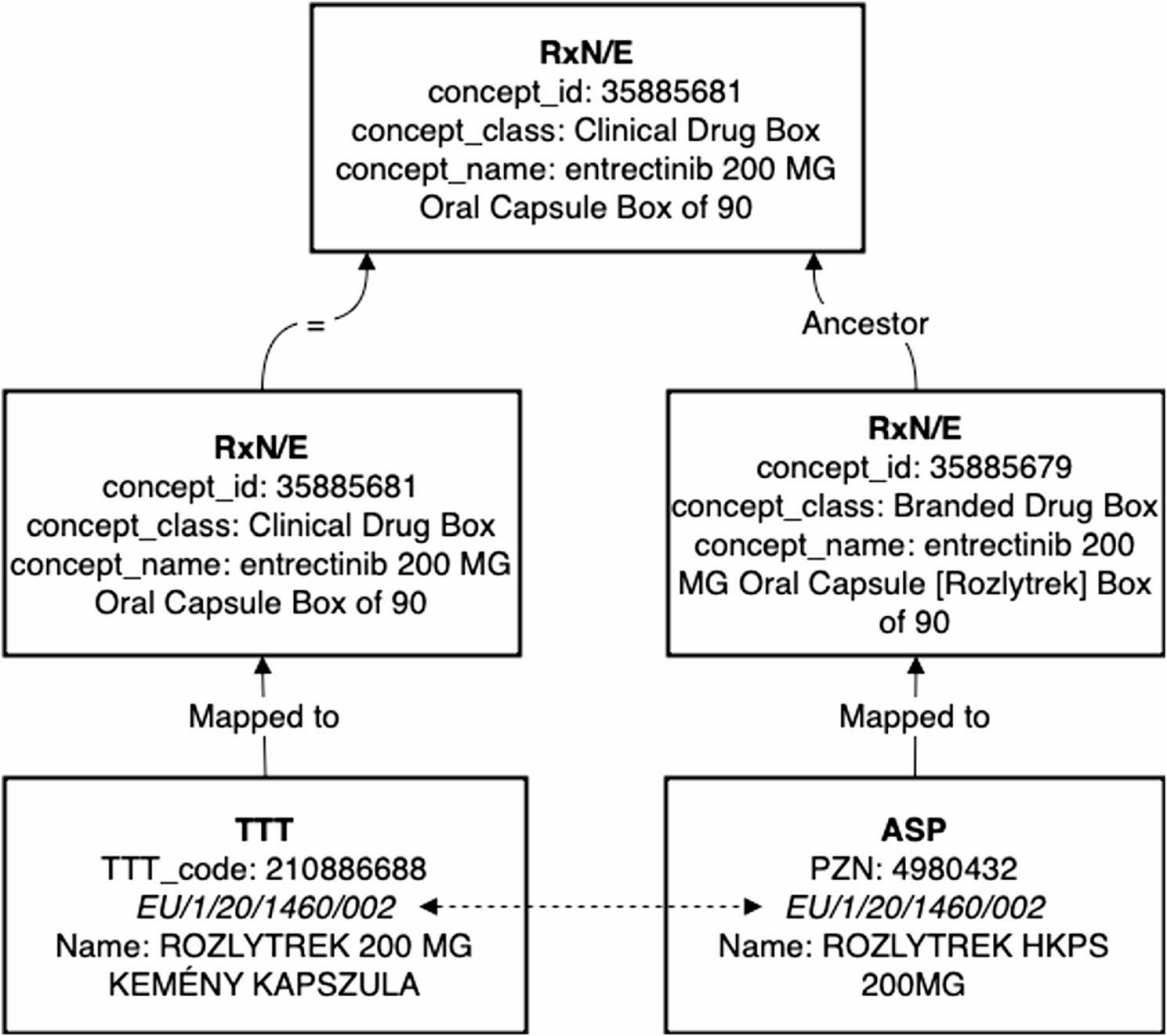
Exemplary concept mappings compared in the validation process. The TTT concept “ROZLYTREK 200 MG KEMÉNY KAPSZULA” was previously to the OMOP RxNorm Extension “entrectinib 200 MG Oral Capsule Box of 90” concept (concept class Clinical Drug Box). The concept “ROZLYTREK HKPS 200MG” from ASP shares the same EU drug registration number with the TTT concept and was assigned by our mapping method to OMOPs RxNorm Extension “entrectinib 200 MG Oral Capsule [Rozlytrek] Box of 90” concept (concept class Branded Drug Box). The shared common ancestor concept here is the clinical drug box concept to which the TTT was mapped

**Fig. 4:**
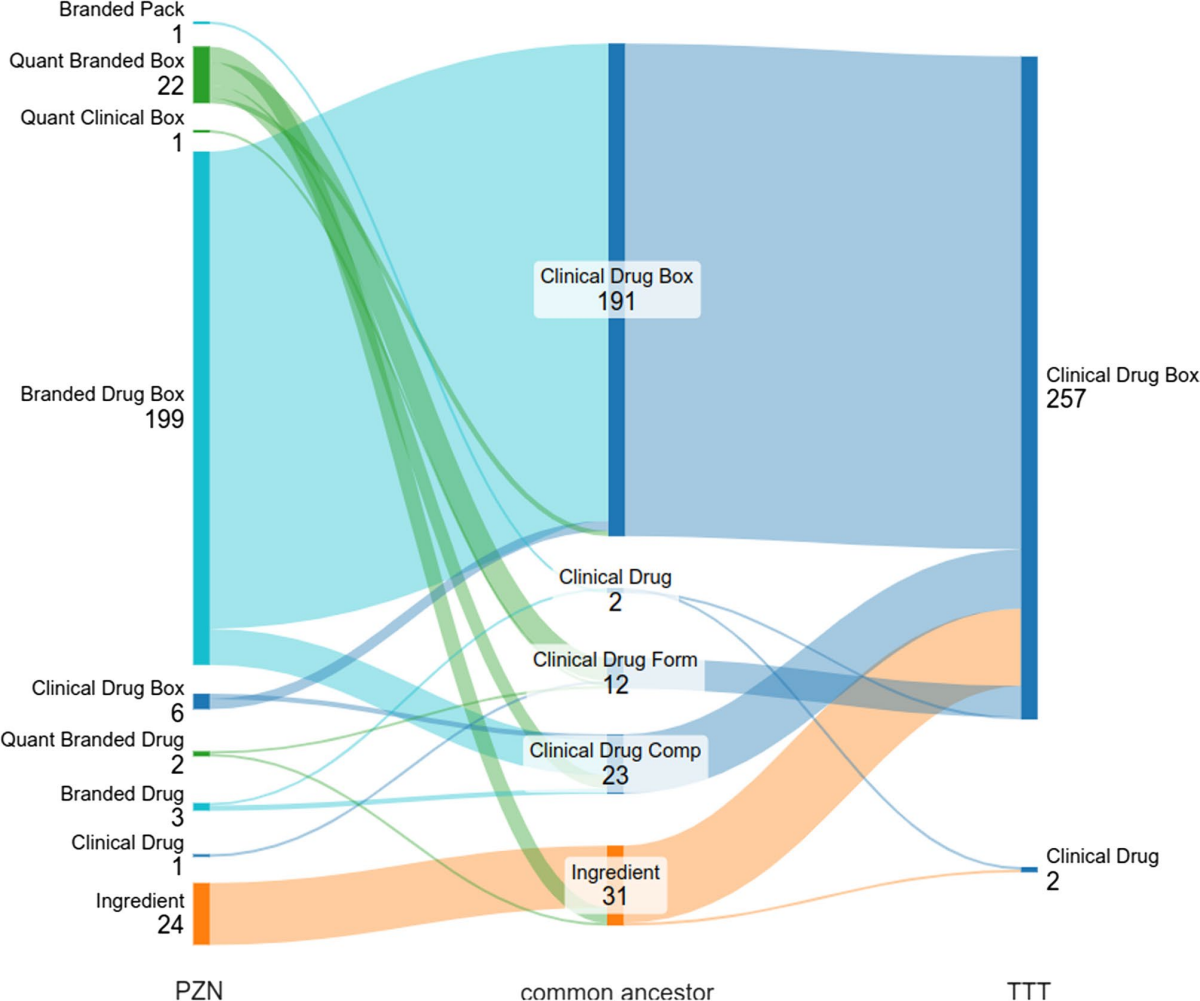
Concept classes and counts for a selection of or mapping results (left column), concepts from the TTT dataset (right column) and concept classes of their shared common RxN/E ancestor concept (middle column). Only concepts that share an EU drug registration number are validated and shown here

The mapping results were validated via the EU drug registration numbers in our source data, together with the TTT dataset. A preprocessing of EU drug registration numbers in ASP was done to resolve duplicated EU drug registration number assignments within the source terminology. We assigned the exact EU drug registration number to PZNs using product information sheets and share this result together with the mapping results [36]. The TTT dataset maps all (480) drugs to OMOP concepts of the class “Clinical Drug”, and the majority are additionally mapped to “Clinical Drug Box” (464) concepts. A total of 260 mappings were selected for validation since they share an EU drug registration number with a PZN concept. Common ancestor concepts were found for all but one mapping, where the associated ingredient concepts were mapped to “dabigatran” vs. the prodrug “dabigatran etexilate”. The majority of common ancestor concepts were of class “Clinical Drug Box” (191 = 73.46%), with the PZN mapping extending the corresponding TTT mappings with brand information. This demonstrates the utility of our mapping approach in deriving reasonable drug concept mappings. An example of such a correspondence is given in Fig. 3. For a graphical summary of all the concept class correspondences identified, see Fig. 4.

### Vocabulary issues

During the process of systematically working with the mentioned terminologies, several challenges and issues were identified, particularly concerning vocabulary inconsistencies and missing pack information. Issues concerning OMOP’s RxN/E vocabularies were systematically documented and communicated through the OHDSI vocabulary GitHub repository^1^. Many of the found inconsistencies concern duplicated concepts and unexpected concept relationships. Further, there is no comprehensive and publicly accessible pack information available within the OMOP RxNorm Extension vocabulary. Pack information, which includes details about the dosage of drug products contained within a pack, is not publicly distributed at the moment^2^. To circumvent this lack of information, we added manual mappings for concepts that needed to be mapped to clinical packs, clinical pack boxes and their branded counterparts based on information contained in the concept names. By feeding discovered vocabulary issues back to the respective communities, we hope to contribute to the overall enhancement of the vocabularies used. 

## Discussion

The proposed mapping of Austria’s national drug terminology to RxN/E via publicly available supplementary data demonstrates the feasibility of using external data in mapping projects and presents a step forward in achieving interoperability and data harmonisation for drug data in Austria. Most previous work concentrated on mappings to RxN/E ingredients only [37]. additionally used dosage information to derive RxN/E drug component mappings. In contrast to earlier work, our approach resulted in a high rate of finer-grained mapping targets, facilitating a more comprehensive integration of drug data across disparate healthcare datasets and thereby, enhancing the secondary use of health data.

By making our mappings available in Usagi CSV and FHIR ConceptMap formats, we aim to enhance transparency and to encourage collaborative refinement of our mappings, which is crucial for maintaining the accuracy and relevance of the mappings over time.

Compared with the European Health Data Evidence Networks (EHDENs) DrugMapping tool [38], our implementation takes package sizes into account and thus is able to derive finer-grained RxN/E mappings for liquid formulation drugs and packaged drugs (beyond the Clinical Drug concept class).

### Challenges and limitations

Primary challenge was the different levels of granularity between the source terminology and RxN/E, especially for dose forms. Our use of probabilistic matching and hierarchical refinement mitigated this issue, but did not eliminate it entirely. Differences in administrable and transformable dose forms further add complexity, a challenge that has been described in [39, 40]. reported poor coverage of dose form concepts between dose forms used in Germany and those provided by EDQM. Ambiguous semantics, resulting in low coder agreement was also mentioned, further underlining the complexity of drug terminology harmonisation.

Manual intervention was necessary for mapping clinical packs and their branded counterparts since OMOP’s pack content information is currently not publicly accessible.

Austria’s national vocabularies are uniquely identifiable via object identifiers (OIDs). For OMOP’s custom vocabularies (e.g. RxNorm Extension) OIDs are currently missing, which complicates referencing a specific vocabulary and its version. Furthermore, the use of semantic web approaches would necessitate the unique identification of vocabulary resources. Representing the source vocabulary in Web Ontology Language (OWL), defining relationships to other vocabularies and deducting mappings could be useful to support the integration of drug vocabularies [5, 13].

Our validation of mappings relies on EU drug registration numbers, which are used at varying degrees of granularity throughout vocabularies. We derived exact EU drug registration numbers for some PZN concepts to be used in the validation process, since those were ambiguously listed in ASP (e.g. EU/16/1157/003–004 vs. EU/16/1157/003). Similar issues were observed in other vocabularies, e.g., the French public drug database (BDPM) [41]. A trusted and centrally managed mapping of drug products with EU drug registration numbers to RxN/E would benefit the European OMOP community.

### Dissemination of results

The dissemination of mapping results is a critical aspect of our approach to ensure transparency, usability, and collaborative refinement within Austria’s health data standardisation community. To facilitate broad access and practical application, we provide the mapping results in two widely recognized formats: Usagi-formatted CSV files and the HL7 FHIR ConceptMap resources. These formats are chosen for their compatibility with existing tools and standards in health informatics, enabling seamless integration into various workflows and systems.

Usagi-formatted CSV files are provided to ensure that the mappings can be readily imported into Usagi for further analysis, validation, and manual adjustments. This format supports ease of use for researchers familiar with OMOP vocabulary management. Furthermore, Usagi can be used to generate *source_to_concept_maps* used in OMOP CDM installations to support vocabulary mapping during OMOP data transformations. By providing our mappings additionally as HL7 FHIR ConceptMap Resources, we enhance the accessibility of our mappings, allowing them to be used in diverse health information systems and facilitating their adoption in various clinical and research settings, e.g. enabling the reuse of mappings in FHIR terminology services [42].

We plan to regularly re-execute our mapping process to reflect changes in the source vocabulary and update the above mentioned resources. Our repository [36] provides a centralised location for accessing the latest version of the mappings. The decision to host the mappings on GitLab, rather than the national terminology server [23], reflects our commitment to ensuring the quality and accuracy of the mappings. Given that these mappings have not yet undergone comprehensive expert review and approval by domain experts, we aim to prevent potential misapplications that might arise from premature publication on official channels. After a level of accuracy and reliability that warrants official publication is achieved, publishing on the national terminology server is desired.

We further actively encourage community feedback to detect misalignments and identify further refinement potentials. By fostering a collaborative approach, we aim to leverage the collective expertise of the community and share the workload of the cumbersome expert validation process among several stakeholders. Mechanisms to convey already approved mappings across re-executions of our proposed mapping process are already implemented. Similarly, the provenance of mappings will also be contained within the provided resources, using Usagi’s status field. This ensures proper attribution of mapping efforts by domain experts.

By providing the mapping results in accessible formats and encouraging community involvement, we aim to create a robust and reliable resource that supports the integration and interoperability of drug data for secondary use of health data in Austria.

### Future work

Efforts to establish a global identification of medicinal products (IDMP) standard will help mitigate the above mentioned mapping ambiguities. The UNICOM project [43] supports the implementation of IDMP at the European level and thus provides valuable harmonisation and integration work. The focus of the European health data space on cross-border electronic prescription and dispensation adds further urgency and justification to this issue. Real world impact on patients’ health and quality of life is demonstrated by the Gravitate-Health project [44], which utilises standardised medicinal product information and health data exchange formats. Austria’s standardisation community is committed to integrate IDMP as a standard to identify medicinal products into the national e-health infrastructure [45]. The development of the IDMP as a suite of International Organization for Standardization (ISO) standards and the involvement of the United States Food and Drug Administration [46] suggests the suitability of this standard to replace RxNorm as standard drug vocabulary within OMOP. Adoption presumed, this could eliminate the need for drug code mappings, in the context of OMOP CDM integrations, altogether.

To ensure broad acceptance of our mapping results, expert validation and feedback are required. Refining mappings in a collaborative manner would ensure their accuracy, reliability and acceptance. A detailed analysis of the characteristics of unmapped drug concepts was not conducted, however we observed that drugs with e.g. herbal ingredients are overrepresented in the portion of unmapped drug concepts. Future work should investigate those unmapped drugs in more detail and analyse mapping results with regards to the most frequently administered drugs. Further, mappings need to be updated whenever source or target vocabularies change. A periodic re-execution of our mapping process along with the source repositories’ ability to track changes will help this cause. The advent of machine learning methods and natural language processing techniques could be explored to refine matching techniques and handle discrepancies in granularity and semantic variations more effectively [47].

Ensuring that the mappings are not only accessible but also integrated into clinical and research-oriented workflows, could be achieved by developing user-friendly interfaces and application programming interfaces. The publication of mapping results on national terminology servers or their integration into OMOP vocabularies needs to be considered.

## Conclusions

This work presents an approach to semiautomatically map a national drug terminology to an internationally recognized drug terminology. Specifically, we mapped Austria’s national drug codes to RxNorm and OMOPs RxNorm Extension. By leveraging publicly available supplementary data, we have demonstrated a feasible approach to develop a semiautomatic mapping process. The traversal of RxNorms concept hierarchy proved to be useful and led to the successful mapping of approximately 95% of the source concepts. In contrast to the traditional mapping to RxNorm ingredients via ATC codes, we were able to derive finer-grained mappings, which include additional information such as dose form, strength and package sizes. The challenges encountered highlight the need for continuous refinement and expert collaboration. With ongoing improvements and broader application, our methodology has the potential to significantly impact health data utilisation for secondary use, ultimately contributing to better healthcare outcomes.

## Supplementary Information

Supplementary Material 1.

## Data Availability

Table 1 provides an overview of supplementary datasets used with indications on how to obtain them. The source vocabulary, ASP, is publicly available from the national terminology server [23]. KHIX2 used as augmentation to the ASP source, is a licensed product and can be obtained from the Austrian Pharmacists’ Publishing House [24]. OMOPs RxNorm and RxNorm Extension is publicly available from the Athena portal [8, 32]. Software resources and mapping results are shared using the MIT licence, via our GitLab repositories (for the archived versions see citation): mapping results [36] (https:/gitlab.com/muv-mim/pzn-rx), mapping scripts [34] (https:/gitlab.com/muv-mim/pzn-rx-matching), Usagi CSV to FHIR [35] (https:/gitlab.com/muv-mim/UsagiCSV-to-FHIR).

## Abbreviations

ASP: German acronym for ‘list of human medicinal products’
ATC: Anatomical therapeutic chemical
BD: Branded drug
BDC: Branded drug component
BDPM: French acronym for the French public drug database
CAS: Chemical abstract service
CD: Clinical drug
CDB: Clinical drug box
CDC: Clinical drug component
CDF: Clinical drug form
CDM: Common data model
CPB: Clinical pack box
CSV: Comma-separated values
CTD: Comparative toxicogenomics database
EDQM: European directorate for the quality of medicines & healthcare
EHDEN: European health data evidence network
EHR: Electronic health record
EMA-PI: EMA product information
EMA: European medicines agency
HIE: Health information exchange
HL7 FHIR: Fast healthcare interoperability resources
IDMP: Identification of medicinal products
ISO: International organization for standardization
NLM: National library of medicine
OHDSI: Observational health data sciences and informatics
OID: Object identifier
OMOP: Observational medical outcomes partnership
OWL: Web ontology language
PZN: German acronym for ‘central drug number’
QBD: Quantified branded drug
QCD: Quantified clinical drug
QCDB: Quantified clinical drug box
RxE: RxNorm extension
RxN: RxNorm
RxN/E: RxNorm and RxNorm extension
SMS: EMA substances management service
TTT: Hungarian acronym for the Hungarian medicine identification numbers
WHO: World health organization

## Footnotes

1
https://github.com/OHDSI/Vocabulary-v5.0/issues/created_by/thisismexp.

2
https://forums.ohdsi.org/t/quantity-of-drugs-within-clinical-branded-packs/18698.
